# Reusability of Scrap Rubber, Tire Shredding, Recycled PVC and Fly Ash for Development of Composites with Vibration Damping Ability

**DOI:** 10.3390/polym16152167

**Published:** 2024-07-30

**Authors:** Dan Dobrotă, Cristinel Sabin Dimulescu, Alin Stăncioiu

**Affiliations:** 1 Department of Industrial Engineering and Management, Faculty of Engineering, Lucian Blaga University of Sibiu, 550024 Sibiu, Romania; 2 S.C. DSC DIMECOSAB S.R.L., 210233 Târgu Jiu, Romania; cristi.dimulescu@yahoo.com; 3 Faculty of Engineering, Constantin Brancuși University of Târgu Jiu, 210185 Târgu Jiu, Romania; stancioiualin09@gmail.com

**Keywords:** composite materials, mechanical tests, vibration amplitudes, frequency response function (FRF), eigenmodes of vibration

## Abstract

The study focuses on harnessing recycled materials to create sustainable and efficient composites, addressing both environmental issues related to waste management and industrial requirements for materials with improved vibration damping properties. The research involves the analysis of the physico-mechanical properties of the obtained composites and the evaluation of their performance in practical applications. Composite materials were tested in terms of their tensile strength and vibration damping capabilities, considering stress–strain diagrams, vibration amplitudes, frequency response functions (FRFs) and vibration modes. The research results have shown that by adding PVC and FA to the rubber-based matrix composition, the stiffness decreases and elasticity increases. The use of FA in the structure of composite materials causes an increase in the vibration damping possibilities due to the fact that it contributes to the chemical properties of the analyzed composite materials. Additionally, the use of PVC results in increased material elasticity, as evidenced by the higher damping factor compared to materials containing only rubber. Simultaneously, the addition of FA and PVC in specific proportions (60 phr) can lead to a decrease in stiffness and a greater increase in the damping factor. The incorporation of PVC and fly ash (FA) particles into rubber-based matrix composites reduces their stiffness and increases their elasticity. These effects are due to the fact that FA particles behave as extensions of chemical bonds during traction, which contributes to the increase in yield elongation. In addition, the use of flexible PVC increases the elasticity of the material, which is evidenced by the increase in the damping factor.

## 1. Introduction

The recycling of rubber, PVC and FA waste into composite materials is an example of a circular economy, where waste is reintegrated into the production cycle, minimizing new waste and maximizing the use of resources. Thus, the use of rubber, PVC and FA waste to obtain composite materials with good vibration damping properties not only contributes to protecting the environment but also contributes to the development of a more sustainable and responsible industry. The use of FA in rubber matrix composites is recommended due to its chemical composition, which is characterized by the presence mainly of silicon (SiO_2_), aluminum (Al_2_O_3_) and calcium (CaO) oxides, along with other chemical compounds in larger quantities. These oxides can interact with the chemical components of the rubber matrix. FA also has a porous surface and may contain hydroxyl groups (–OH) on the surface, which can form hydrogen bonds or other types of chemical interactions with rubber polymers. Damping is an important property of systems and has a significant impact on their dynamic behavior. This can be caused by various mechanisms such as internal friction, air resistance or other energy losses. Damping in a system has the role of limiting oscillations and preventing unwanted resonance phenomena. Resonance is a physical phenomenon in which a system or structure vibrates or oscillates with an increased amplitude at a certain excitation frequency. According to those presented in [1,2], rubber matrix materials are frequently used for vibration attenuation in various structures due to their specific mechanical properties. The behavior of rubber can be classified into two main categories: hyperelastic and viscoelastic. Rubber is a viscoelastic material. At a low strain, it is linear viscoelastic in nature, but at a high strain, the rubber behaves as a hyperelastic body, which means it behaves as a non-linear viscoelastic material. In the linear viscoelastic regime, the elastic modulus of a material is independent of strain. The dynamic properties of rubber matrix composites allow for very good vibration isolation and damping characteristics. For example, by adding large amounts of carbon black, an increase in the damping capacity of the rubber can be achieved by decreasing the transmissibility of vibrations at the resonance frequency. Carbon black improves both the damping capacity and stiffness of rubber matrix composites through complex interactions with polymer chains and the mechanical load distribution. The optimization of the concentration and manufacturing processes is essential to obtain composites that effectively combine these properties. The amount of carbon black added to the rubber matrix needs to be optimized. Too high of a concentration can increase the stiffness at the expense of the flexibility and damping capacity. Typically, concentrations between 20% and 40% are used to find a balance between stiffness and damping. Methods for obtaining carbon black from biological sources, such as agricultural residues or biomass, are being developed to reduce the environmental impact. These carbon black alternative materials offer diverse options for various industrial and commercial applications while ensuring that performance needs are met in a more sustainable and environmentally friendly way. But the increased levels of carbon black also cause an increase in the stiffness of the compound and the resonance frequency [3,4].

Dynamic mechanical analysis (DMA) can be used to determine the impact vibration damping of phenyl silicone rubber. Analyses of how the sizes of the rubber particles obtained from tire shredding influence the vibration damping behavior have been carried out. The results indicated that if large rubber particle sizes are used, the vibration damping performance can be improved. Hysteresis vibration damping involves absorbing and dissipating mechanical energy as heat during charge and discharge cycles. Larger rubber particles can contribute to more effective hysteresis, as their uniform distribution allows for better interaction with the matrix and more efficient energy dissipation [5,6].

Previous research has focused on the use of bamboo biochar (BB) as a filler in natural rubber (NR) composites. In a study, it was determined that sound transmission loss (STL) can be improved by adding 10–20 phr BB. The introduction of BB in the NR matrix positively influences the vibration damping, physical, mechanical and thermal properties [7].

The vibration damping properties of an elastomer can be improved by non-regenerable carbon black. Due to the fact that non-renewable carbon black cannot be obtained through sustainable processes, it is important to use new materials instead. The use of residues from the food industry can be a sustainable option for replacing carbon black. It is very important, however, that the evaluation of the vibration damping performance of these composites is directly evaluated for each type of part, because it can depend on their geometry [8,9]. EPDM rubber (Ethylene–Propylene–Dien Monomer) is a type of synthetic rubber used in various applications due to its elastic properties and resistance to environmental factors. The behavior of this rubber is generally hyperelastic, which allows it to behave appropriately at large deformations and stretches [10]. Many researchers have developed mathematical models to describe the hyperelastic behavior of rubber. EPDM is characterized by good damping properties and can be used when vibration isolation or a reduction in vibration transfer is required [11]. In [12], the natural frequencies for sandwich structures were determined as follows:

- Variant 1: structural steel sandwiched between EPDM rubber (the structural steel is placed between two layers of EPDM rubber; this arrangement can be used to isolate vibrations or to reduce their transmission from the steel to other components of the structure);
- Variant 2: EPDM rubber sandwiched between structural steel plates (in this configuration, the EPDM rubber is placed between two structural steel pieces; this arrangement can be used to absorb or dampen vibrations and protect the steel structure against impacts or harmful vibrations).

In [13], the damping properties of the asphalt mixture were analyzed, in which rubber particles were introduced to replace part of the fine aggregate, after which they were mixed with acrylonitrile-modified polymers (AMPs). As the content of rubber particles increases, the stiffness of the RPMAP (rubber particle-modified acrylonitrile polymer) decreases, the energy loss factor increases, and the damping properties improve. As the content of rubber particles increases, the material no longer has the ability to convert kinetic energy into elastic potential energy. The effect of magnetic rubber powder (DRM) on the damping properties was analyzed by Mei and co-workers [14]. Two methods were chosen to fix the MRP on a vibrated steel beam: one using magnetic attraction (called DRM beam) and the other using adhesive bonding (called AB-MRP beam). The study authors developed a formula to predict the damping characteristics of the MRP beam, which was experimentally validated. These results can be used to determine the advantages and limitations of MRPs.

In [15], a new type of rubber–sand concrete (RSC) was proposed, which has a composition consisting of rubber, sand and cement. During the study, the effects of the three components of the RSC composition on the following properties were discussed: density, Poisson’s ratio, elastic modulus, compressive and tensile strengths, energy dissipation capacity, dynamic shear modulus and damping ratio. Thus, research has shown that very good damping properties can be obtained for RSC-type materials if an optimal composition is chosen for them. The typical composition of composites with good damping properties may include varying proportions, for example, 10–20% granulated rubber, 60–70% sand or aggregate and 15–25% cement. This composition can vary depending on the application, from pavements and platforms to industrial floors and noise structures. Rubber, sand and cement composite materials are recognized for their ability to absorb vibrations and noise due to the porous structure and energy-absorbing capacity of rubber. The damping properties are influenced by the relative proportions of the components and the degree of porosity of the material.

In the course of the research in [16], a new type of magnetic rubber, obtained by adding Fe_3_O_4_ nanoparticles to a natural rubber matrix, was analyzed, and then, the authors demonstrated that such a material can have good damping properties. The result is due to the fact that after applying a magnetic field, the magnetic nanoparticles on the rubber matrix are magnetized; at the same time, magnetic dipole moments are induced, which causes a magnetic field and can absorb the shock energy.

The research in [17] deals with a number of aspects related to the use of an elastomeric nanocomposite for constrained layer damping (CLD) arrangements to reduce structural vibrations with a low weight. The reuse of used tires is very important, and in this sense, in [18], the influence of the dynamic shear deformation, γ, on the shear modulus, G, and the damping ratio, D, of soils mixed with shredded rubber resulting from waste was analyzed for tires.

In [19], the effect of surface treatment of natural rubber with bis (3-triethoxysilylpropyl) tetrasulfide (TESPT) was investigated. The results showed that the incorporation of TESPT into natural rubber allows for obtaining a very good rubber damper. In [20], a novel approach was presented to estimate the Young’s modulus of a functional gradient rubber composite (FGRC) made of cap with and without (FA). In search of a lightweight elastomeric vibration damping material, the research in [21,22] focused on the fabrication of two nanocomposites obtained from a 50/50 mixture of nitrile rubber/polyvinyl chloride. Likewise, in [23], the effect of using TiO_2_/NBR-PVC fibers, which were obtained by spinning the NBR casting solution mixed with PVC and nano-titanium dioxide (TiO_2_), was analyzed on the damping properties and the mechanical properties of NBR-PVC hollow fibers loaded with TiO_2_. All these studies demonstrate the fact that composite materials with rubber matrices can be a very good technical solution for obtaining parts with very good damping properties.

Thus, the research presented in this paper was focused on the analysis of the possibility of obtaining composite materials containing rubber particles obtained by grinding tires, PVC and FA, respectively, that have good damping properties. In this sense, five types of samples were made with different compositions of PVC and FA rubber, respectively.

## 2. Materials and Methods

### 2.1. Materials Used in Research

As part of the research, composite materials were manufactured with rubber matrices that had, in their structure, a series of additions of regenerated rubber as well as rubber powder obtained from shredding tires, FA and, respectively, PVC. In order to be able to carry out an analysis of the influence of each compound in the composite material on the damping properties, 5 samples of composite materials were manufactured, containing a mixture of recovered rubber and rubber powder, which was obtained by recycling and grinding waste tires (S01) that had, in their composition, recovered rubber + rubber powder, which was obtained by recycling and grinding of waste tires. Starting from the composition of sample S01, new composite materials were made by adding PVC (S02), FA (S03), PVC and FA in moderate amounts (S04) and PVC and FA in very large amounts (S05), respectively. The compositions of the analyzed materials are presented in Table 1.

The determination of the compositions of the 5 types of samples was made considering the need to obtain composite materials with a rubber matrix that perform as well as possible in terms of their damping capacities. In the case of sample S01, a proportion of 50 phr (regenerated rubber + rubber powder) material, resulting from the grinding of waste from tires, was used, and this proportion gradually increased, reaching, as in the case of sample S05, 150 phr (regenerated rubber + PVC + FA).

Natural rubber SIR-20 was chosen because it is a versatile and economical category of natural rubber, suitable for a wide range of industrial applications, from tires to various technical products, having very good elastic properties and low production costs. The choice of SBR-1723 TDAE rubber was made because it is a very versatile and durable type of synthetic rubber widely used in industrial applications. Its properties of wear resistance, elasticity and chemical stability make it ideal for obtaining products with vibration damping properties. The addition of poly-butadiene synthetic rubber SKD ND in the composition of the materials was made because it offers very good properties of elasticity, wear resistance and excellent dynamic performance, which thus make it ideal for applications that require flexibility and vibration damping. Reclaimed rubber was obtained from waste tires, and its use was considered to be an economic and ecological alternative to new rubber that is suitable for a wide range of industrial applications, and although it may have some limitations in terms of its mechanical and chemical properties, its economic and environmental benefits make it an attractive option in many contexts. The use of rubber powder with a size of 80–100 µm in the composition of the materials was decided due to its low cost and ecological benefits, but also due to the fact that it allows for improved vibration damping properties and durability of the products containing such material. The coupling agent KWQ-EL has a composition based on hexadecyltrimethoxysilane (HDS).

#### 2.1.1. Characteristics of the Used FA Powders

FA was obtained from M/s Energetic Oltenia Complex, Romania. Since FA with various properties can result from the combustion of lignite, 10 samples were taken (using a glass container with a volume of 100 mL; the samples were chosen from 10 different places), which were analyzed from the point of view of granulation and their chemical composition. Also, the FA used for the analyses was initially subjected to a grinding and sieving process to improve the granulation.

The FA particle size significantly influences the mechanical properties and damping efficiency of rubber matrix composites. A fine and uniform distribution of FA particles can improve the mechanical strength, vibration damping capacity, sound insulation and durability of the composite material. Precise particle size control and the use of advanced dispersion techniques are essential to obtain composite materials with optimized performances for various practical applications.

FA has a complex chemical composition, which can significantly influence the properties of composite materials containing PVC or rubber. The chemical composition of fly ash mainly includes oxides of silicon (SiO_2_), aluminum (Al_2_O_3_), iron (Fe_2_O_3_) and calcium (CaO) along with other elements and compounds in smaller amounts. The appropriate choice and treatment of FA can optimize the performance of these composites, providing improvements in their mechanical, thermal, chemical and physical properties, and this allows for their use in a wide range of industrial applications that must provide vibration damping.

The FA used in the research was initially analyzed using optical microscopy to establish the polydispersity (OLYMPUS BX 51M Optical Microscope, Olympus, Bartlett, IL, USA). The results obtained from the polydispersity analysis were subsequently processed using the analysis of variance (ANOVA). To estimate the average particle size of FA, 100 particles were measured, and the number average size *D_n_*, gravimetric average size *D_w_* and polydispersity index (*D_w_*/*D_n_*) were determined:

(1) $$D_{n}=\frac{\sum_{i}N_{i}\cdot D_{i}}{\sum_{i}N_{i}}$$

(2) $${\;D}_{w}=\frac{\sum_{i}N_{i}\cdot D_{i}^{4}}{\sum_{i}N_{i}D_{i}^{3}}$$

(3) $$PDI=\frac{D_{w}}{D_{n}}$$

where *N_i_* is the number of particles that have size *D_i_*. Based on the average numerical size *D_n_* of the particles and, respectively, the average gravimetric size *D_w_*, the value of the polydispersity index was calculated:

*PDI* = 0.917 (4)

The value of *PDI* determined using the relationship indicates a homogeneous dispersion of particle sizes. To establish the particle size distribution, their sizes were measured using laser diffraction using a Diffraction Microtrac MRB laser manufactured by 215 Key-stone Dr. Montgomeryville, PA 18936, USA. FA was observed to have a distribution generally in the range of 2–9 μm. Most of the particles had a diameter of about 5 μm.

Adjusting the size of the PVC particles through grinding techniques or raw material selection can allow for the optimization of the properties of the composite materials for specific applications. Thus, the particle size distribution of PVC plays a crucial role in determining the overall properties and performance of rubber matrix composites. A fine and uniform distribution of PVC particles can significantly improve the mechanical properties, vibration damping capacity, thermal and acoustic insulations, processability and durability of the composite material. Precise particle size control and the use of advanced dispersion techniques are essential to obtain composite materials with optimal performances in various practical applications. Smaller PVC particles can disperse vibrational energy more effectively, contributing to better vibration damping. This is beneficial for applications where noise and vibration reductions are desired. As for large PVC particles, they tend to be less effective in dispersing vibrational energy, which can lead to a reduced damping capacity.

X-ray analysis of the FA was used to establish the phases, which was performed with the X-ray diffractometer DE X’Pert PRO MPD, PANALYTICAL. The FA used in the research was composed of quartz, mullite (or porcelainite) and hematite. In order to better observe the properties of FA, an analysis of its chemical composition was also carried out, and the results, methods and equipment used are presented in Table 2.

#### 2.1.2. Characteristics of the PVC Particles Used in the Research

For the experiments, they were purchased from S.C. CRILELMAR S.R.L Targu-Jiu, Romania. The PVC granules obtained from recycled materials using an EREMA-type granulator.

The granules obtained from recycled PVC were transformed into fine material particles by grinding and sieving. The particle size of the PVC was analyzed using optical microscopy (Olympus B 51XM Optical Microscope), and the values obtained were processed using the analysis of variance (ANOVA). After measuring the particle sizes (using the OLYMPUS B 51XM optical microscope equipped with STREAM ESSENTIALS image analysis and measurement software) and calculating the numerical size *D_n_*, and respectively, the average gravimetric size *D_w_*, the value of the polydispersity index was determined:

*DI* = 0.901 (5)

The physical–mechanical properties of PVC particles, according to the specifications in the specialized literature, are presented in Table 3.

The size of the PVC particles can influence the final properties of the composite material. The uniform particle size proves not only the uniform manufacture but also better plasticizing properties. The influence of the microscopic granular structure on the processing is ascertained by determining the volumetric weight, either by casting, by settling or by sieving. By sieving the dry polymer, it is possible to quantitatively determine both the particle size and the degree of dispersion, establishing, in this way, both the average value of the particle size per assortment and the average of the general degree of dispersion. At a higher dispersion, small particles are found in the voids between the large granules, which increase the volumetric weight determined by settling. Therefore, apart from the average diameter of the particles, there is another factor that influences settling, namely the density of small, isolated particles, which is higher when the particles are hard and have a smoother surface. The optimal shape is presented by a material whose particles have a medium size, a low degree of dispersion and a low volumetric weight (determined by settling). Regarding the PVC particles, they were characterized from the point of view of polydispersity according to the values presented in the case of FA. Thus, it was established that the size of the PVC particles used had values in the range of 0.5 to 1 mm.

### 2.2. Samples Manufacturing Technology

Obtaining composite materials with a rubber matrix involved several technological operations, but the first operation consisted in the homogenization of the materials used. This homogenization operation was applied to all the samples obtained, even in the case of the sample without FA and/or PVC additions, so that all the components that entered the structure of the materials were as well distributed as possible. The materials were homogenized in a mixer with the help of the mixer model LS-S130 with a double helical paddle for 2.5 min. Five batches of the mixture were prepared for the five types of specimens. The batch was homogenized with a two-roll mill. In order to obtain a miscible mixture of regenerated rubber, powder rubber, fly ash and PVC waste, it was necessary to add a compatibilizing additive, namely the coupling agent KWQ-EL, and thus, the adhesion between the components was improved to ensure the formation of a homogeneous structure. The coupling agent used helps to reduce the interfacial tensions and increase the cohesion between the different phases of the composite.

The establishment of the working regimen was made, taking into account the indications given in [25]. The working temperature of the cylinders was adjusted to 80 °C. The ratio between the peripheral speeds of the two cylinders was set at 1:1.10. The batch processing time was set at 10 min/batch. In order to avoid a long rolling, which reduces the thermal stability of the material, the processing was carried out in small batches in relation to the capacity of the roll. This had to be taken into account, especially since waste was used. After batch homogenization, the next processing step was calendering. Using the calendering process, 2 mm to 10 mm thick sheets were prepared. In our case, having the small batches only for experiments, we chose that the processing of the mixture sheets at the established thickness should also be conducted on the roller by adjusting the distance between the cylinders.

**Figure 1:**
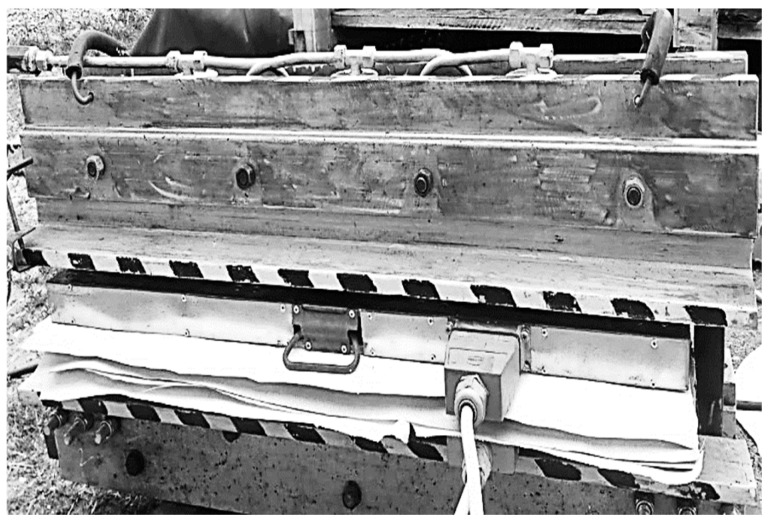
Mobile hot vulcanizing press, type DSLQ.

Subsequent to homogenization and calendering, the composite sheets were vulcanized in a mobile vulcanization press of the DSLQ type, produced by Wagener Schwelm Corporation (Reisholzstraße, Hilden, Germany). This mobile press is shown in Figure 1. The main constructive characteristics of the DSLQ vulcanizing press are as follows: it is compact and reliable; the heating of the samples is conducted uniformly over the entire surface; the pressure is evenly distributed over the entire vulcanized surface; it has a low electricity consumption, high thermal efficiency, constant temperature and vulcanization time control.

The vulcanizing press can be used to vulcanize all types of rubber conveyor belts, belts with textile inserts, steel cable belts, technical plates, PVC belts, etc. The mobile vulcanizing press can be used in metallurgy, mining, power plants, ports and places where there are no explosive or corrosive gases.

The choice of this type of vulcanizing press was made because it allows for the vulcanization parameters (pressure and temperature) to be adjusted in a very wide range. The adjustment of the vulcanization parameters was made, so that there was a correlation of the pressure with the vulcanization temperature as follows:

- For the temperature in the range 30 °C–50 °C: pressure = 6.8 MPa;
- For the temperature in the range 50 °C–70 °C: pressure = 8.3 MPa;
- For the temperature in the range 70 °C–100 °C: pressure = 10.5 MPa;
- For the temperature in the range 100 °C–125 °C: pressure = 13.0 MPa;
- For the temperature in the range 125 °C–140 °C: pressure = 15.5 MPa.

**Figure 2:**
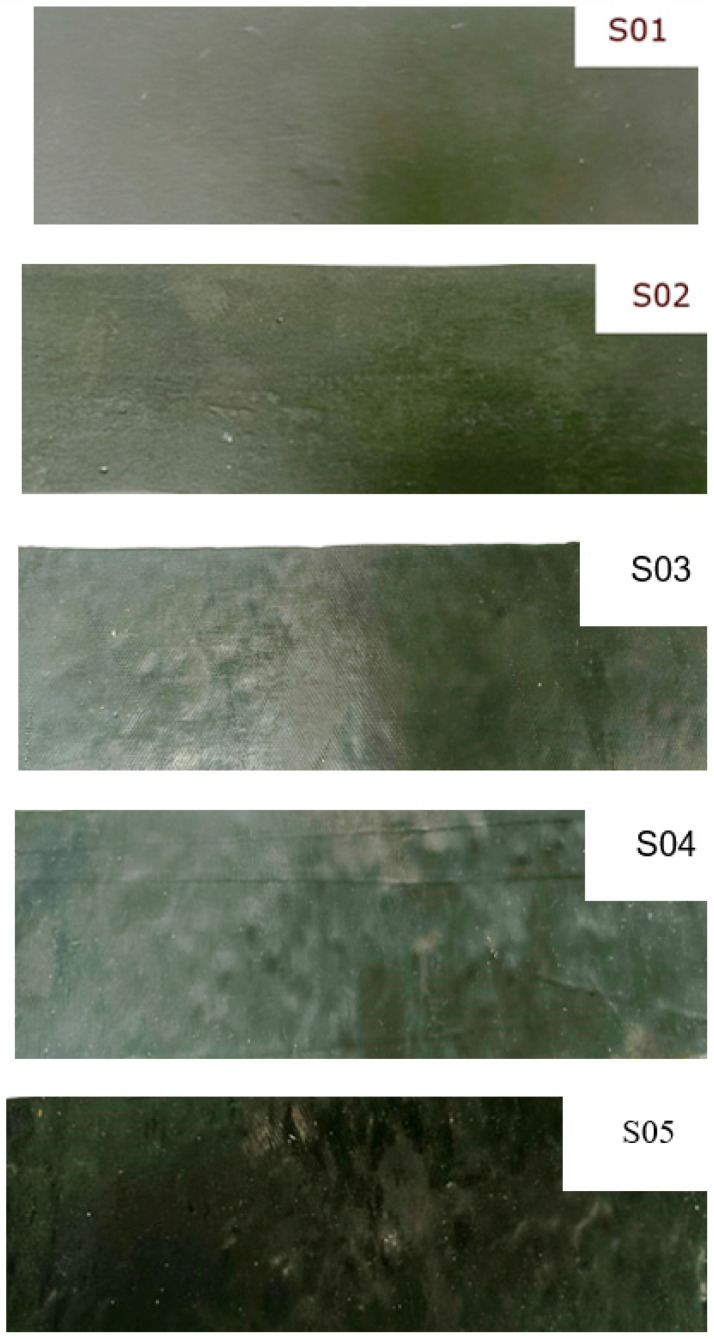
Samples of types S01, S02, S03, S04 and S05.

The vulcanization was conducted at 125 °C for 50 min under 15.5 MPa pressure, and subsequently slowly cooled to 50 °C under the same pressure. The samples obtained are shown in Figure 2.

It was necessary to cool the samples after vulcanization under pressure to avoid the formation of pores in the structure of the materials. The cooling rate of rubber matrix composites has a significant impact on the microstructure and mechanical properties of the final material. Precise control of this parameter is essential to obtain materials with optimized performances for various practical applications. Slower cooling can lead to a more uniform distribution of fillers and improved mechanical properties.

### 2.3. Theoretical Aspects Related to Modal Identification in Vibration Damping Processes

The equations of motion for a mechanical system with ‘n’ degrees of freedom, loaded by a system of external excitations, can be represented using a system of ordinary differential equations of the second order. These equations are typically derived using Newton’s second law of motion and take into account the forces, displacements and velocities of ‘n’ mass points connected by elastic elements of stiffness ‘k_k_’ and damping elements of the coefficient of depreciation ‘c_k_’.

The general form of the equations of motion for such a system can be written as:

M · x″(t) + C · x′(t) + K · x(t) = F(t)(6)

where the following are true:

- M is the mass matrix, which is a symmetric matrix of size nxn that represents the masses m_k_ of the n mass points;
- x(t) is a vector of generalized displacements for the n degrees of freedom according to time t;
- x″(t) is the second derivative of x(t) as a function of time, representing the vector of generalized accelerations;
- C’ is the damping matrix, which is also a symmetric matrix of size nxn, usually diagonal, which represents the damping coefficients ck for each degree of freedom;
- x′(t) is the first derivative of x(t) as a function of time, representing the vector of generalized velocities;
- K is the stiffness matrix, which is a symmetric matrix of size n × n that represents the stiffness coefficients kk for each degree of freedom;
- F(t) is the vector of external forces and excitations applied to the system according to time t.

The solution of this system of differential equations gives the time-dependent behavior of the degrees of freedom of the system x(t) in response to the external excitation F(t). The specific shape and values of the mass matrix M, the damping matrix C, the stiffness matrix K and the external excitation F(t) depend on the details of the modeled mechanical system. These matrices and vectors are typically determined by the system geometry, material properties and boundary conditions. After these matrices and the external excitation are defined, numerical methods or analytical techniques, such as the finite element method or modal analysis, can be used to solve these equations and obtain the dynamic response of the system.

The mathematical representation of the system response to an external excitation using a modal analysis approach can be expressed as a sum of n modal contributions due to each degree of freedom:

(7) $$\left\{X(\omega )\right\}=\sum_{k=1}^{N}\left[\frac{\left\{\psi^{k}\right\}\cdot {\left\{\psi^{k}\right\}}^{T}\cdot \left\{Q(\omega )\right\}}{a_{k}(-\mu_{k}+i(\omega -\nu_{k}))}+\frac{\left\{\bar{\psi^{k}}\right\}\cdot {\left\{\bar{\psi^{k}}\right\}}^{T}\cdot \left\{Q(\omega )\right\}}{{\bar{a}}_{k}(-\mu_{k}+i(\omega +\nu_{k}))}\right]$$

where the following are true:

- {*X*(*ω*)} represents the Fourier transform of the displacement; in the frequency domain, it describes how the system responds to external excitation at a certain frequency;
- $\left\{\psi^{k}\right\}\;\mathrm{and}\;\left\{{\bar{\psi }}^{k}\right\}$ are the eigenvector and its complex conjugate corresponding to the mode *k* of the system; the eigenvectors describe the shape or form of the response mode of the system;
- *μ_k_* is the damping factor corresponding to module *k* of the system;
- *ν_k_* is the natural frequency of mode *k*;
- *a_k_* and ${\bar{a}}_{k}$ are the normalization constants of the eigenvector; these constants are often used to scale or normalize the modal shapes;
- *ω* is the external excitation frequency.

In order to obtain a correct model corresponding to practical applications, the modal vectors were replaced by two modal constants, as defined by Equations (8) and (9).

(8) $$\frac{\psi_{i}^{k}\cdot \psi_{j}^{k}}{a_{k}}=U_{ij}^{k}+i\cdot V_{ij}^{k}$$

(9) $$\frac{{\bar{\psi }}_{i}^{k}\cdot {\bar{\psi }}_{j}^{k}}{{\bar{a}}_{k}}=U_{ij}^{k}-i\cdot V_{ij}^{k}$$

Using the notations in Equations (8) and (9), we can define the admittance of the system with Equation (7) as the ratio between the strain response and the excitation force:

(10) $$\alpha_{ij}\left(\omega \right)=\sum_{k=1}^{n}\left[\frac{U_{ij}^{k}+i\cdot V_{ij}^{k}}{-\mu_{k}+i\cdot \left(\omega -\nu_{k}\right)}+\frac{U_{ij}^{k}-i\cdot V_{ij}^{k}}{-\mu_{k}+i\cdot \left(\omega +\nu_{k}\right)}\right]\;$$

Obtaining the mathematical model was achieved by considering a series of approximations but also using the concept of a discrete system with the mass concentrated in n material points. The mathematical model presents the following particularities:

- Discrete system with concentrated mass: the mathematical model approximates the real system as a discrete system, with the mass concentrated in ‘n’ material points; this means that the system is represented by a set of discrete points, with the mass concentrated at those points;
- Large value of n for precise approximation: to obtain a precise approximation of the real system with the discrete system, it is desirable that ‘n’ has a large value; this implies that a large number of material points are required to represent the system accurately; however, practical limits, including experimental techniques and time restrictions, may prevent the use of an excessively large n value;
- Limited frequency range: the frequency range is limited to some reasonable value; this limit is analyzed by the resonance limit of the equipment and the objectives that characterize each application;
- Sum reduced to a few components: due to the limited frequency domain, the sum in Relation (5) is reduced to a smaller number of components;
- Correction factors: two correction factors are introduced, the “lower modal admittance” for the lower modes and the “residual flexibility” for the higher modes; these factors take into account the contributions of the different modes of vibration in the system;
- System admittance: the overall system admittance is expressed and is assumed to incorporate the effects of both lower and higher modes, presumably to account for system response characteristics.

In the approximations made to create the mathematical model, the concept of a discrete system with masses concentrated in ‘n’ material points was used. For an accurate approximation of the real system by the discrete system, ‘n’ must have a large value. This is not possible due to experimental limitations and processing techniques, as well as the time required for data processing. In applications, the frequency range is limited to a reasonable width determined by the major resonances of the equipment being analyzed and the frequency range specific to the purpose of the application. Under these conditions, the sum in Relation (10) is reduced to a few components, also denoted by ‘n’.

The following notations were made:

- Lower modal admittance: $-\frac{1}{M_{ij}^{\prime }\omega^{2}}$;
- Residual flexibility: $S_{ij}^{\prime }$.

With these notations, Equation (5) takes the following form:

(11) $$\alpha_{ij}\left(\omega \right)=\frac{-1}{M_{ij}^{\prime }\cdot \omega^{2}}+\sum_{k=1}^{n}\left(\frac{U_{ij}^{k}+i\cdot V_{ij}^{k}}{-\mu_{k}+i\cdot \left(\omega -\nu_{k}\right)}+\frac{U_{ij}^{k}-i\cdot V_{ij}^{k}}{-\mu_{k}+i\cdot \left(\omega +\nu_{k}\right)}\right)+S_{ij}^{\prime }$$

With the aforementioned details, it can be said that the modal identification of a system with *n* degrees of freedom requires the determination of 4n modal parameters: *μ_k_*, *ν_k_*, *U_jl_^k^* and *V_jl_^k^* (these parameters are intrinsic characteristics of the system and describe the way the system responds to different types of excitations). Modal parameters are important, because they remain independent of external conditions, and changes in these parameters can help identify problems such as wear or cracks in the structure.

Using the determined modal parameters, the response of the system to different types of excitations can be calculated. The most common types of excitations for which the response can be calculated are as follows:

- Seismic movement: this refers to vibrations or ground movements caused by earthquakes; understanding how a structure responds to seismic motion is crucial to seismic engineering and structural safety;
- Concentrated electrodynamic forces: these forces are related to switching phenomena;
- Distributed forces due to the action of the wind: wind can exert distributed forces on a structure; determining how these forces affect the structure is important for evaluating its wind resistance and safety.

Modal parameters are determined using experimental tests. To do this, the system is brought into a state of controlled vibration. Various methods of low-level excitation are mentioned to achieve this state of controlled vibration, including the following:

- Loading the system gradually with a force: this method involves applying a gradual force to the system in a controlled way to induce vibrations;
- Single-point sinusoidal or broadband vibrational excitation: this method involves the application of sinusoidal vibrations at a single point or over a range of frequencies to study the response of the system;
- Impact force: this method involves applying an impact force to the system, which induces vibrations and allows the system response to be measured.

The impact force method was used in the research. Based on this model, the modal parameters were identified for the samples studied.

### 2.4. Identification of Modal Parameters for Composites with Rubber Matrices and FA and PVC Particles, Respectively

**Figure 3:**
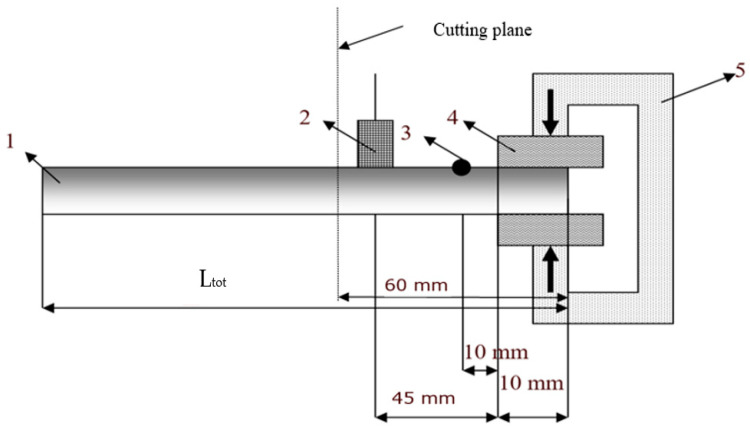
Experimental set-up to identify the eigenmodes of vibrations. 1—analyzed sample; 2—accelerometer; 3—excitation point; 4—rubber plates; 5—clamping vise.

For the modal identification, the experimental assembly from Figure 3 was used. L_tot_, the total length of the sample in the console, was 65 mm, the sample width 6 mm and the thickness 6 mm; these samples were obtained by cutting from initial samples with a length of 200 mm, a width of 50 mm and a thickness of 6 mm; the mass was obtained by weighing these initial samples.

The following equipment was used:

- Accelerometer with a sensitivity of 0.04 (Produced B&K, Yorba Linda, CA, USA) connected to a NEXUS-type signal conditioner (manufacturer B&K);
- Impact hammer with a sensitivity of 1.020, produced by B&K Yorba Linda, CA, USA, connected to a NEXUS-type signal conditioner;
- Signal conditioner connected to SPIDER 8-type data acquisition system, Phoenix, Blomberg, Germany;
- SPIDER 8 data acquisition system connected via USB port to the notebook, Phoenix, Blomberg, Germany;
- Interface software between SPIDER 8 and notebook called CATMANEASY, Phoenix, Blomberg, Germany.

Note: at a distance greater than 60 mm from the mounting point, the vibrations were dampened, and the accelerometer recorded only the background noise.

### 2.5. Determination of Tensile Strength of Composite Materials

For the tensile test, an Instron 1000 HDX universal testing machine equipped with BlueHill 3 software was used to determine the experimental data in real time but also to control the machine and process the results. The Bluehill 3 program allows for the following actions: automatic sensor calibration; generation of predefined and user-edited reports; system monitoring; viewing results in real time; the possibility of determining conventional and real characteristic curves and plasticity characteristics. For the tensile test, flat prismatic specimens with the following dimensions were used: 250 mm long, 25 mm wide and 10 mm thick. To cut the samples and to make their side surfaces as uniform as possible, the Labotom 5 hand-operated cutting machine was used. The cutting of the samples was necessary, because after vulcanization, plates of sizes much larger than the size of the samples were obtained.

## 3. Results and Discussion

The use of composite materials with rubber matrices and additions of FA or PVC can represent an optimal solution for the production of parts with the role of vibration damping. The combination of PVC and FA rubbers can provide improved vibration damping properties compared to traditional materials. The rubber matrix provides excellent cushioning, while the additions of FA and PVC can contribute to improved stiffness and strength. In order to be able to improve the damping characteristics, an adjustment of the composition and the volume fraction of FA or PVC is necessary. The research carried out was focused on the development of advanced composite materials with a rubber matrix and PVC and FA additions with improved vibration damping properties. Thus, the aim was to find a versatile solution for the production of effective vibration dampening parts. This approach can present challenges associated with vibration control in various applications.

### 3.1. Determination of Vibration Amplitude

The vibration amplitude of rubber matrix composite materials is influenced by several factors related to the material composition, structural design and dynamic conditions. The design and geometry of the composite material structure also play an important role. The interaction between the various components and the overall structural design can affect how vibrations are transmitted and absorbed. The damping ratio, which quantifies the energy dissipation capacity of a material, is an important parameter. Rubber matrix composite materials aim to have a high damping ratio to effectively reduce vibration amplitudes.

**Figure 4:**
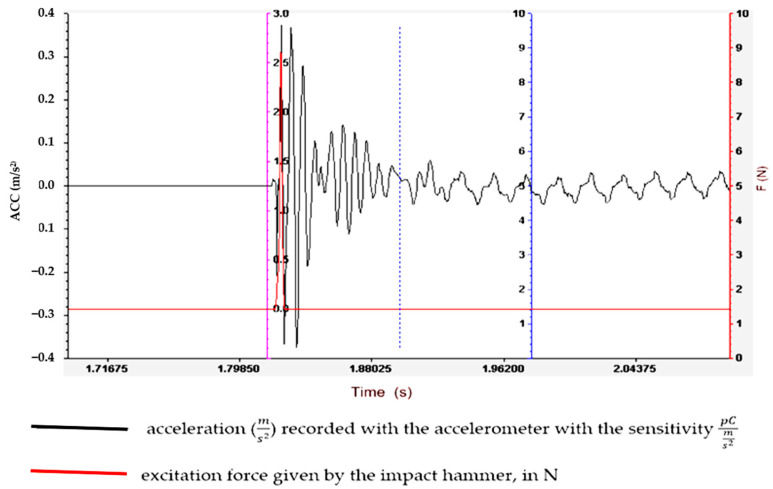
Experimental vibration records for sample S01.

**Figure 5:**
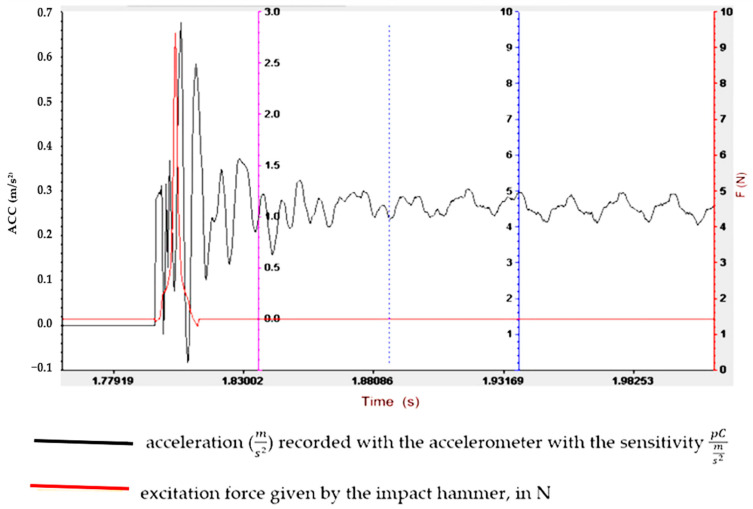
Experimental vibration records for sample S02.

**Figure 6:**
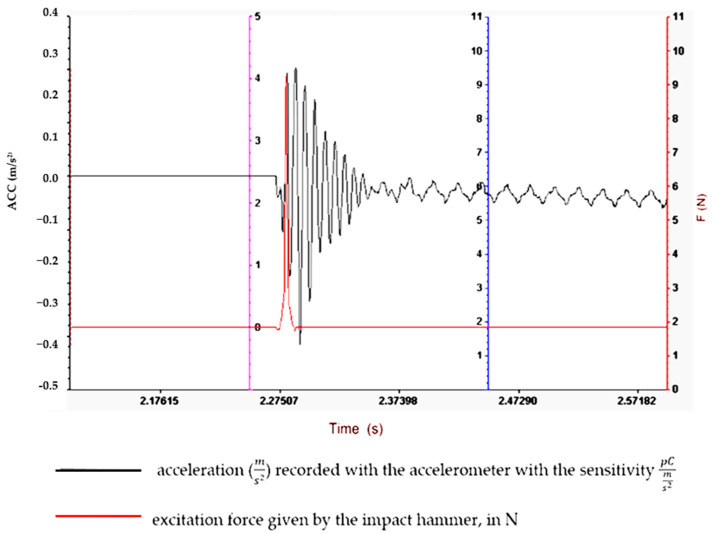
Experimental vibration records for sample S03.

**Figure 7:**
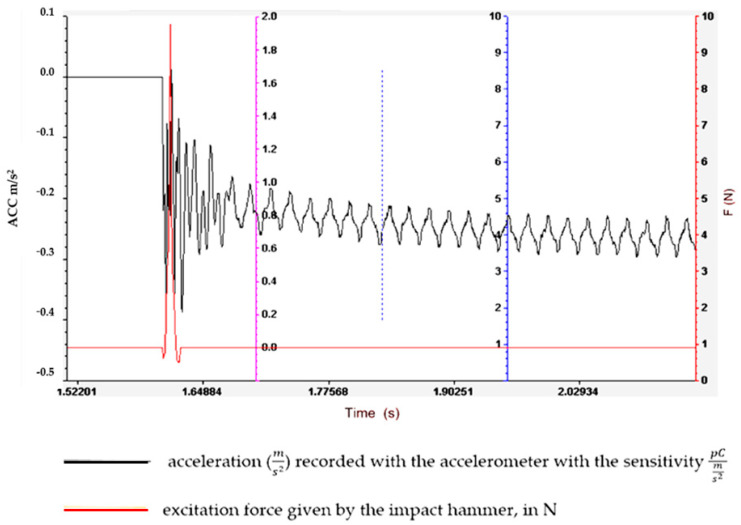
Experimental vibration records for sample S04.

**Figure 8:**
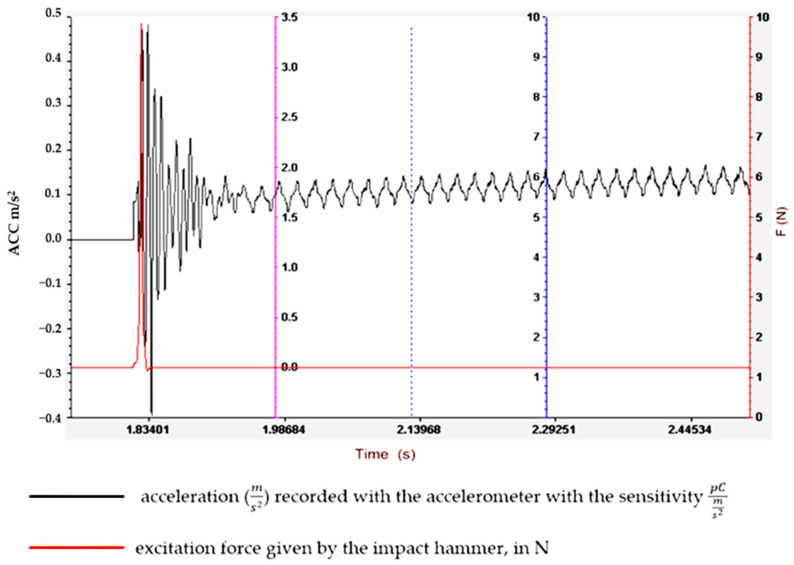
Experimental vibration records for sample S05.

Understanding and optimizing these aspects can aid in the design of rubber matrix composite materials that exhibit the desired vibration damping performance for specific applications. It often involves a balance between the stiffness, strength and damping characteristics to meet the operational requirements. To determine the vibration amplitude for samples S01–S05, the experimental set-up shown in Figure 3 is used, and the results obtained are shown in Figure 4, Figure 5, Figure 6, Figure 7 and Figure 8.

Regarding the lowest vibration amplitude, it was recorded in the case of sample S04, which has quite large amounts of PVC (30 phr) and FA (30 phr) in its composition (Figure 6). Also, the fastest vibration damping was observed in the case of specimen S05, which contains the highest amounts of PVC and FA (Figure 7). These results confirm that the addition of FA and PVC in a composite material with a rubber matrix can considerably change the damping properties due to the modification in the main factor of the homogeneity of the composite structure [26,27].

### 3.2. Determination of the Frequency Response Function (FRF)

The frequency response function is related to the transfer function of the system. The resonance peaks in the FRF indicate the natural frequencies of the structure, and the width of these peaks provides information about the damping.

**Figure 9:**
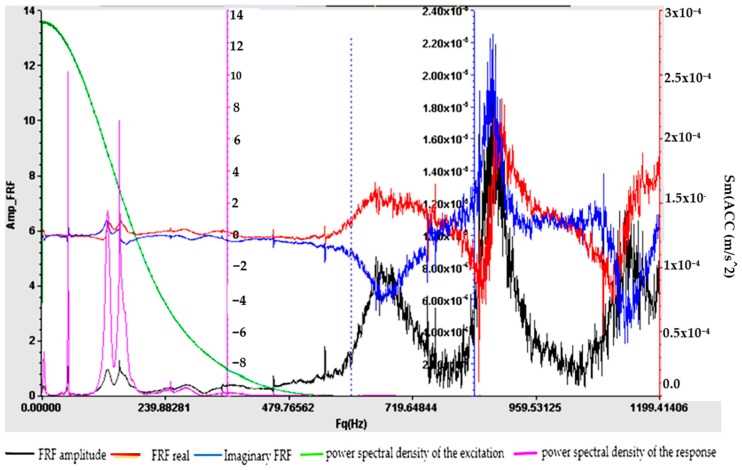
Frequency response function (FRF) for S01.

**Figure 10:**
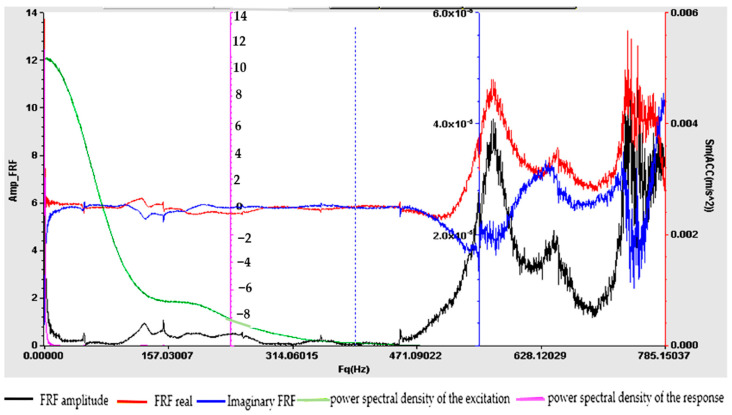
Frequency response function (FRF) for S02.

**Figure 11:**
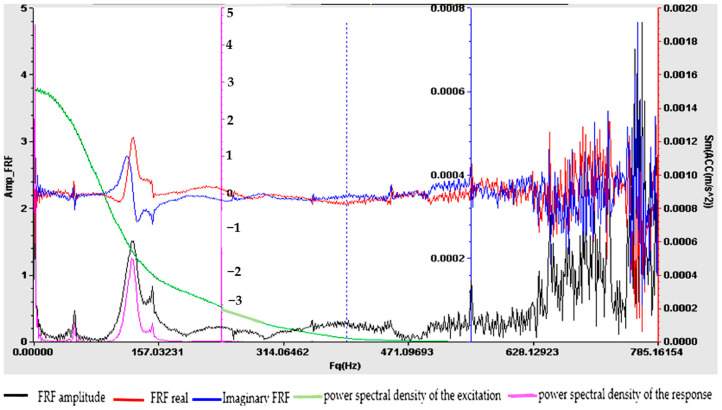
Frequency response function (FRF) for S03.

**Figure 12:**
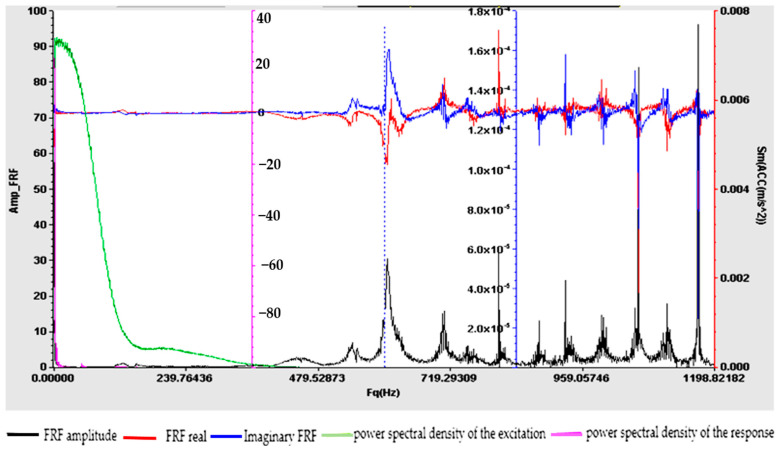
Frequency response function (FRF) for S04.

**Figure 13:**
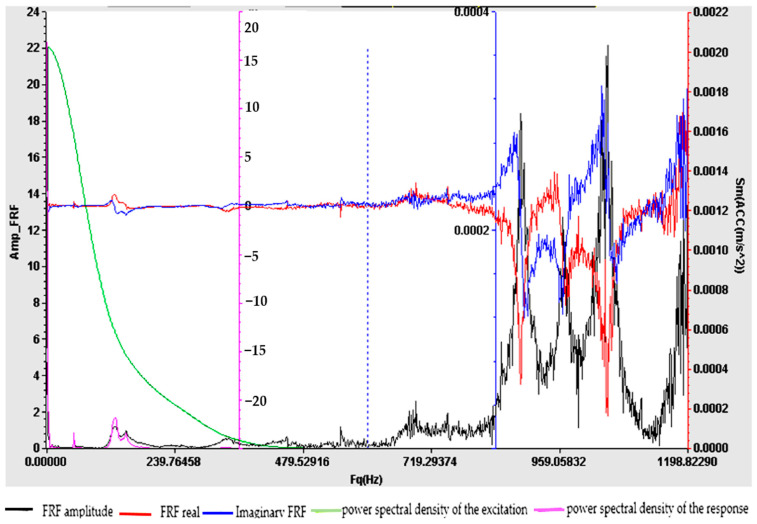
Frequency response function (FRF) for S05.

Understanding the frequency response function is crucial for designing structures, evaluating their dynamic behavior and ensuring that they operate within acceptable vibration limits. The experimental determination of FRF is a valuable tool for structural health monitoring, modal analysis and vibration control applications. For samples S01–S05, the frequency response function (FRF), roughly defined as the ratio between the Fourier transforms of the response and the excitation, was calculated. Figure 9, Figure 10, Figure 11, Figure 12 and Figure 13 show the frequency response characteristics in Cartesian coordinates.

The frequency response function (FRF), being a critical parameter in structural dynamics and vibration analysis, can provide us with information on how the materials in samples S01–S05 behave under dynamic stress. The peaks in the FRFs correspond to the natural frequencies, while the phase information indicates the phase relationship between the input and the output. The results obtained and presented in Figure 9, Figure 10, Figure 11, Figure 12 and Figure 13 demonstrate the fact that the addition of PVC and, respectively, FA causes a substantial change in the frequency response function (FRF).

### 3.3. Determination of Eigenmodes of Vibration

Determining the appropriate vibration modes, also known as mode shapes, is a crucial step in structural dynamics and modal analysis. Modal analysis is the process of experimentally or analytically determining the natural frequencies, mode shapes, and damping ratios of a structure. Experimental modal analysis involves measuring the response of a structure to identify its dynamic characteristics.

Eigenmodes of vibration influence how stresses are distributed and dissipated in the material. Composites with more eigenmodes can distribute stresses more evenly, reducing the risk of stress build-up and structural failure. Research and experimental studies use modal analysis to identify the eigenmodes of vibration of composites. The number of natural modes of vibration significantly influences the performance and practical applications of rubber matrix composites. By optimizing these modes, materials with tunable damping and stiffness properties suitable for a wide range of industrial, automotive, medical and protective applications can be created.

The accurate determination of vibration modes is essential for understanding the dynamic behaviors of structures, identifying potential resonance problems and designing effective vibration control strategies. Modal analysis provides valuable information about the natural frequencies and mode shapes that govern a structure’s response to dynamic excitations.

**Figure 14:**
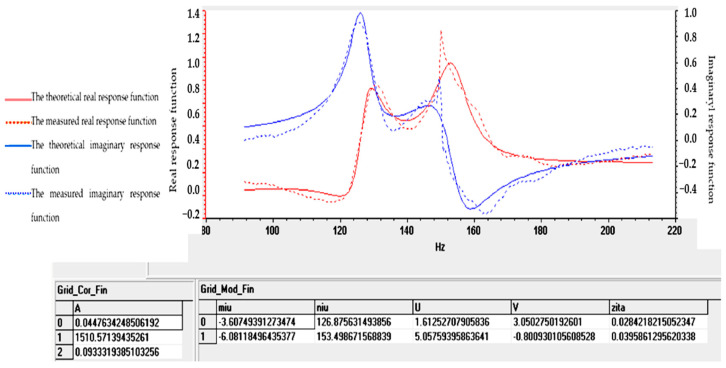
Determination of vibrational eigenmodes for S01.

**Figure 15:**
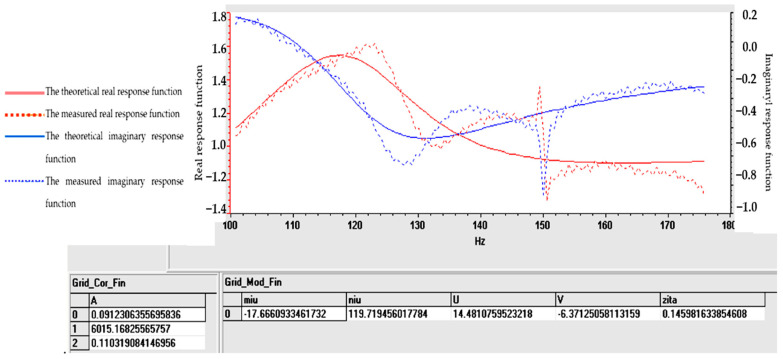
Determination of vibrational eigenmodes for S02.

**Figure 16:**
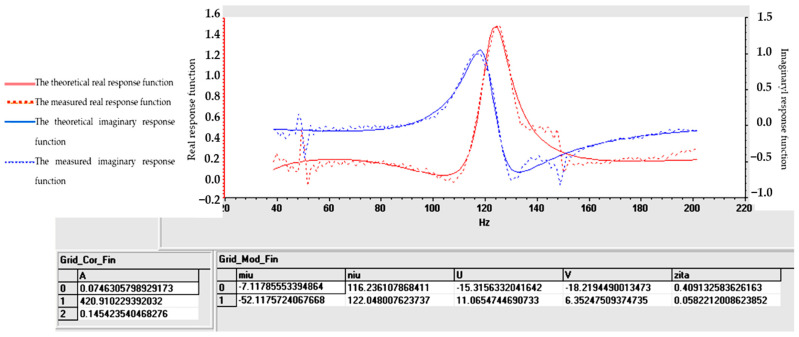
Determination of vibrational eigenmodes for S03.

**Figure 17:**
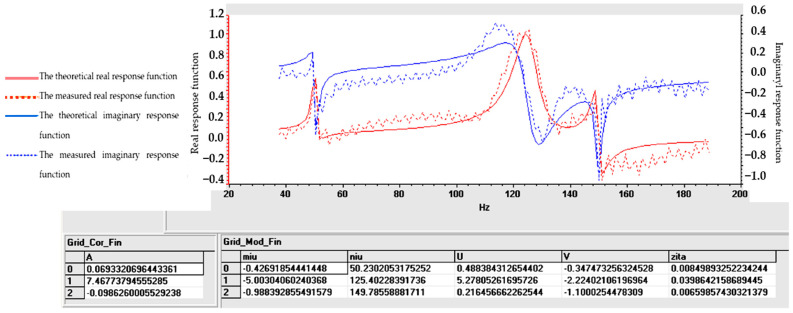
Determination of vibrational eigenmodes for S04.

**Figure 18:**
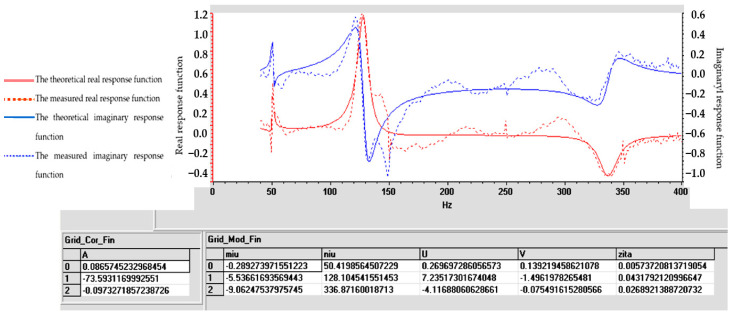
Determination of vibrational eigenmodes for S05.

Tables and graphs of the natural vibration modes of specimens S01–S05 are presented in Figure 14, Figure 15, Figure 16, Figure 17 and Figure 18.

The notations in Figure 14, Figure 15, Figure 16, Figure 17 and Figure 18 are as follows: (μ) is the vibration damping factor per mass unit; (ν) is the natural frequency or natural frequency; U and V define the real and imaginary parts; (ζ) is critical damping; 0, 1, 2..... are the identified vibration modes.

Note: The minus sign in Figure 14, Figure 15, Figure 16, Figure 17 and Figure 18 shows the fact that the damping has the role of opposing the vibratory movement, and that is why it was considered with the “−” sign in the table. Since the vast majority of specialized papers [10,11] consider this parameter to be treated with the “+” sign, it will be referred to with this sign hereinafter.

From the analysis of the results in Figure 14, Figure 15, Figure 16, Figure 17 and Figure 18, the following trends were observed:

- Between one and three proper modes of vibration were determined, depending on the analyzed sample;
- For sample S01, natural frequencies were determined in the range of 126–153 Hz, and the damping factor (known in the specialized literature as the damping factor per mass unit) corresponding to the first natural mode was 3.607 (Ns/m/kg);
- For sample S02, a single natural mode of vibration was identified at the frequency of 119 Hz, having a damping factor of 17,666 (Ns/m/kg);
- For sample S03, natural frequencies were determined in the range of 116–122 Hz, and the damping factor (known in the specialized literature as the damping factor per mass unit) corresponding to the first natural mode was 7.117 (Ns/m/kg);
- Samples S04 and S05 have close natural frequencies, and the damping factors for the first natural mode of vibration are approximately equal.

It is known that there is a direct proportionality between the dynamic stiffness and the natural frequency, thus [28]:

(12) $$E\cdot I=39.478418\cdot \rho \cdot g\cdot w\cdot {\left(\frac{\nu \cdot l^{2}}{\vartheta }\right)}^{2}$$

The same is found related to the dynamic longitudinal modulus of elasticity (Relation (12) [28]).

(13) $$E=38.32\cdot \rho {\left(\frac{\nu \cdot l^{2}}{g}\right)}^{2}$$

Another parameter that can be determined from the vibration analysis is the energy loss factor (“loss factor”), with the Relation (14) [28].

(14) $$\eta =0.3183099\cdot \frac{\mu }{\nu }$$

The vibration (energy) loss factor is a measure of how quickly energy is dissipated from a vibrating system during oscillations. The loss factor is an important characteristic for evaluating the dynamic behaviors of objects, including structures, machines, components and systems. The dissipation factor is often denoted by “η” and can vary between 0 and 1. A dissipation factor of 0 indicates that there is no energy dissipation, meaning that the oscillations will continue without damping out. A loss factor of 1 indicates that all energy is dissipated in each cycle, so the oscillations stop quickly.

The loss factor can be influenced by several factors, including the material the object is constructed from, added damping mechanisms (such as shock absorbers), geometry and how the system operates. A higher loss factor indicates stronger damping of oscillations, which may be desirable in certain applications, such as machines or structures exposed to vibrating forces. Conversely, a lower loss factor may be desired in some applications, such as resonance in electromechanical devices, where we want to minimize power dissipation.

If we refer to the damping factor per unit length C (Ns/m/m), then it can be determined with Relation (15) [28].

(15) C=2·μ·ρ·g·w

In Relations (12)–(15), the following notations were made: *ρ* is the density of the material, *g* is the thickness of the material, *w* is the width of the material, *l* is the free length of the sample (from the console), and *ϑ* is a constant that is determined from the support conditions of samples (sample embedded at one end and free at the other) and has a value of 1.875 [29]. By applying Relations (12)–(15), the results in Table 4 were obtained.

From the analysis of the results in Figure 14, Figure 15, Figure 16, Figure 17 and Figure 18, from a dynamic point of view, the following trends can be deduced:

- Sample S01 has the highest dynamic stiffness it is marked by the high value of the natural frequency, in the context in which all samples were analyzed with the same geometric dimensions and the direct proportionality between stiffness and frequency was taken into account; this phenomenon was preserved as in the case of static tensile stress or Shore A hardness analysis);
- Sample S02 has a lower stiffness compared to S01 but a higher damping factor (i.e., the sample becomes more elastic); this phenomenon was preserved as in the case of static tensile stress or Shore A hardness analysis;
- Sample S03 has a lower stiffness compared to S01 but a higher damping factor (i.e., the sample becomes more elastic); this phenomenon was preserved as in the case of static tensile stress or Shore A hardness analysis;
- If a comparison is made between samples S02 and S03, a better behavior of sample S02 (with 30 phr FA) is observed compared to S03 (with 30 phr PVC) both in terms of its stiffness and its elasticity (absorbs vibrations better); a possible explanation for this phenomenon is the fact that although the PVC particles are elastic (PVC is flexible and has a good elongation at break following tensile stress–axial stress); after hitting the samples with the impact hammer, they vibrate in the plane vertically (flexural vibration), and the PVC particles have a different behavior in bending compared to traction (they tend to deform without fully returning to their original shape, which leads to a decrease in elasticity following vibrations in the vertical plane);
- In samples S04 and S05, large decreases in the stiffness but also in the damping factor are observed, which make them usable in areas where there must be no energy dissipation, which means that the oscillations will continue without attenuation.

### 3.4. Experimental Results Obtained from the Tensile Test

For the tensile test of the specimens made from the five types of composite material, a test method was developed in the proper language of the Instron-type testing machine, namely Bluehill 3. The following parameters were established at this stage: the type of test (tensile), material data (specimen shape, specimen width and distance between machine trays), test speed, machine limits, machine acquisition rate (10 points/s), output file type (ASCII or DIF: Data Interchange Format, a file format that can be picked up by any of the statistical data processing programs) and type of output data to be collected. Regarding the test speed, it was set at 50 mm/min.

**Figure 19:**
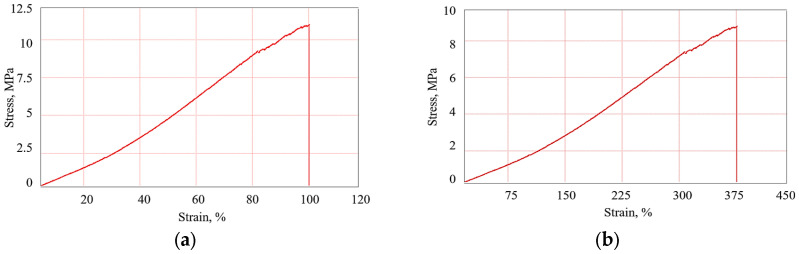

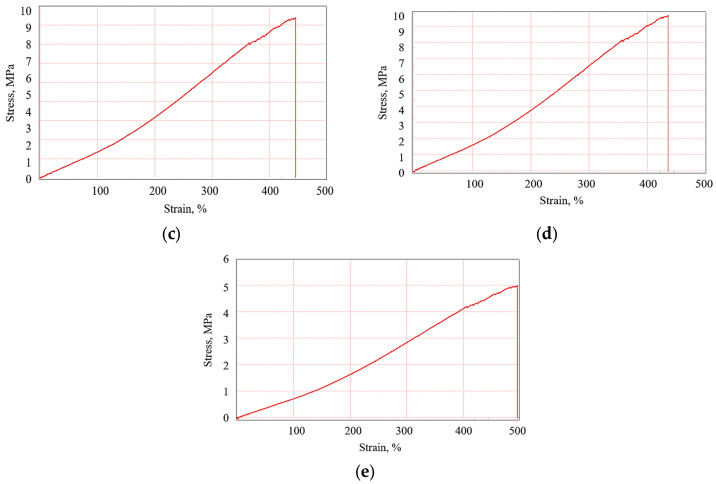
Stress–strain curves for the tested samples: (**a**) sample S01; (**b**) sample S02; (**c**) sample S03; (**d**) sample S01; (**e**) sample S01.

The results obtained for all five samples analyzed are presented in Table 5, and the characteristic stress–strain curves in Figure 19. The results in Table 5 represent the arithmetic mean of 15 test specimens/sets of material.

Thus, it was found that if FA is introduced into the rubber matrix there is an increase in the elasticity (highlighted by the values of elongation at break of the specimens) with a decrease in the breaking strength. One explanation for this phenomenon is that the FA particles inserted into the matrix, at the granular level, can act as extension elements of the rubber granules due to the impurities they contain, allowing the whole structure to elongate more (a similar explanation was given in the specialized literature in [25,28]). The addition of PVC led to an increase in the elasticity (since PVC is characterized by high elongation at break) with a decrease in the strength (since PVC is characterized by low breaking strength in comparison with that of S01 rubber).

Thus, it has been shown that the addition of PVC can cause a better distribution of mechanical loads in the composite. This contributes to the reduction of stress concentration and, thus, to an improved mechanical performance. The addition of PVC and FA in rubber matrix composites can have beneficial effects on their mechanical strength. Research has shown that PVC contributes to increased stiffness and tensile strength, while FA improves interfacial adhesion. The combination of these materials can lead to the development of durable composites with superior mechanical properties suitable for various industrial applications.

From the analysis of the results obtained from the vibration tests and the tensile tests, it could be observed that PVC can help to attenuate high-frequency vibrations due to its inherent damping properties. However, the overall effect depends on the balance between stiffness and flexibility in the composite. Also, due to increasing the stiffness of the composite by adding PVC, the damping capacity of low-frequency vibrations can be reduced. In these conditions, it is essential to optimize the proportion of PVC to maintain an appropriate balance between vibration damping and stiffness. FA, on the other hand, can enhance the energy absorption capacity and improve vibration damping over a wide range of frequencies due to its uniform density and dispersion.

The combination of these materials must be carefully optimized to obtain composites with excellent vibration damping properties and suitable mechanical performances for various industrial applications. Often times, optimizing one set of properties (e.g., tensile strength) can negatively affect other properties (e.g., damping). For example, a very stiff matrix may improve the tensile strength but reduce the damping capacity. Under these conditions, through the conducted research, an attempt was made to design composite materials that would allow for the balancing of these properties.

In conclusion, the relationship between the tensile strength and damping properties of composite materials is influenced by numerous factors that must be carefully balanced to achieve the desired performance in specific applications. Continued research in this area is essential for the development of composite materials with optimized performances.

## 4. Conclusions

From the point of view of dynamic mechanical properties, the following can be observed:

- The addition of PVC and FA particles in composite materials with rubber-based matrices leads to a decrease in their stiffness and an increase in their elasticity; this can be explained by the fact that the FA particles behave, during traction, as extensions of chemical bonds and contribute to the increase of the flow elongation; also, since flexible PVC was used, it contributed to increasing the elasticity of the material, a fact realized by increasing the damping factor compared to sample S01;
- The addition of PVC and FA in rubber matrix composites can have beneficial effects on their mechanical strength. Samples S04 and S05 have low vibration damping properties as well as low energy loss factors; consequently, these materials can be used for structures where the oscillations must continue without attenuation;
- The research allowed us to establish the fields in which the analyzed samples can be used. Thus, the samples S02 and S03 can be successfully used for the following types of applications: car suspensions and shock absorbers for cars: in the car industry, it is important to ensure a moderate level of vibration damping to provide passenger comfort and improve road grip; energy dissipation values between 0.01 and 0.05 can be considered ideal to achieve a balance between comfort and chassis control;
- Constructions and buildings: in the design of building structures or bridges, it is important to take into account the damping of vibrations generated by wind, earthquakes or other sources; energy dissipation within said range helps protect the building and occupants against excessive damage during seismic events or extreme weather conditions;
- Industrial machines and equipment: industrial machines and equipment can be subject to different levels of vibrations; in many cases, a moderate level of energy dissipation is necessary to avoid damage to machines and to maintain their performance; this applies, for example, to processing machinery, conveyors, production equipment and other industrial machinery;
- Medical devices: medical devices, such as ultrasound or medical imaging equipment, must be stable and have a certain vibration damping capacity in order to obtain accurate images or to ensure safe medical procedures;
- High-performance electronics: in high-performance electronics, such as particle accelerators or laboratory equipment, it is important to minimize vibrations to ensure accurate measurements or the correct operation of devices; however, excessive damping could disrupt the operation of such equipment.

The areas in which samples S04 and S05 can be used are as follows:

- The construction of some elements of electronic radio oscillators: they must work at precise frequencies; in this case, minimizing power dissipation is crucial to ensure circuit stability and efficiency;
- The design of some transformer elements: resonance in a transformer can lead to increased energy losses in the form of heat and reduced energy conversion efficiency; by designing the transformer to minimize resonance and power dissipation, its efficiency can be improved;
- Elements that make up MEMS sensors (Micro-Electro-Mechanical System): these sensors can be sensitive to resonance, which can affect their measurements; to obtain accurate results, it is important to minimize power dissipation and avoid resonance in these devices;
- Elements that make up piezoelectric actuators: piezoelectric actuators are used in applications such as lens-focusing devices in cameras or in medical devices for the precise delivery of medicines; to maintain accuracy and control, it is essential to minimize power dissipation and avoid resonance.

As far as future research is concerned, we will pursue the production of new composite materials that have very good damping properties specific to each field of use. Also, in future research, we will pursue the development of new composite materials made from waste that provide a better balance between tensile strength and damping.

## Figures and Tables

**Table 1:** Compositions of the materials used to obtain the samples.

Materials	The Compositions of the Materials Used, phr				
	S01	S02	S03	S04	S05
Natural rubber	15	15	15	15	15
Styrene–butadiene synthetic rubber (SBR-1723 TDAE)	20	20	20	20	20
Poly-butadiene synthetic rubber SKD ND (Nizhnekamsk, Russia)	15	15	15	15	15
Reclaimed rubber (ARTEGO, Targu Jiu, Romania)	30	30	30	30	30
Rubber powder with size of 80–100 µm (ARTEGO, Romania)	20	20	20	20	20
Carbon black HAF 330	18	18	18	18	18
Vulcanization accelerator DPG	4	4	4	4	4
Coupling agent KWQ-EL (ARTEGO, Romania)	5	5	5	5	5
Sulphur	3	3	3	3	3
Fly ash (Energetic Complex Oltenia, Targu Jiu, Romania)	0	30	0	30	50
Polyvinyl chloride (Crillelmar SRL, Targu Jiu, Romania)	0	0	30	30	50
Total	130	160	160	190	230

**Table 2:** Chemical composition of FA, and, respectively, the methods and equipment used.

Chemical Composition	Content (% Mass)	Method	Equipment
Silica (SiO_2_)	52.97	X-ray fluorescence (XRF)	X-ray analytical microscope, XGT 9000; the fluorescent X-ray detector can detect from americium down to carbon using a light element detector, produced by Horiba, Kyoto 601-8510, Japan
Aluminum oxide (Al_2_O_3_)	19.41		
Ferric oxide (Fe_2_O_3_)	1.27		
Calcium oxide (CaO)	4.38		
Magnesium oxide (MgO)	4.57		
Potassium oxide (K_2_O)	4.56		
Sodium oxide (Na_2_O)	4.01		
Titanium dioxide (TiO_2_)	3.83		
Sulfur trioxide (SO_3_)	2.07		
Loss on ignition—LOI	2.93		

**Table 3:** Physico-mechanical properties of PVC particles [24].

Material Type	Mechanical Properties				
PVC particles	Resilience, [J/m]	Young’s modulus [GPa]	Tensile strength [MPa]	Elongation at break [%]	Poisson’s ratio
	550	1.4	16	180	0.39

**Table 4:** Dynamic mechanical characteristics.

Parameters	Samples				
	S01	S02	S03	S04	S05
*ρ* [kg/m^3^]	570.33	533.83	517.5	470	445
m [g]	34.22	32.03	31.05	28.2	26.7
EI [N·m^2^]	0.483	0.403	0.368	0.062	0.075
E [MPa]	174	140	137	22.33	21.14
η	9.057 × 10^−3^	0.047	0.019	2.674 × 10^−3^	1.85 × 10^−3^
C [Ns/m/m]	3.798	17.41	6.807	0.365	0.305

**Table 5:** Experimental results of the mechanical properties for the analyzed samples, obtained after tensile stress.

S01	S02	S03	S04	S05
σ_r_ = 11.6 [MPa]	σ_r_ = 8.6 [MPa]	σ_r_ = 9.2 [MPa]	σ_r_ = 9.91 [MPa]	σ_r_ = 5.11 [MPa]
A = 100 [%]	A = 375 [%]	A = 444 [%]	A = 423 [%]	A = 498 [%]

## Data Availability

The original contributions presented in the study are included in the article, further inquiries can be directed to the corresponding author.

## References

[1] Prasada V.B.S.R., Rao G.V., Idrees M. (2020). Identification of Damping Characteristics of EPDM-RUBBER with applications to sandwiched beams and considerations to Engine Mounts for Performance Evaluation. Mater. Today Proc..

[2] Naidu B.M., Prasad V.R., Rao D.G.V. (2016). On The Evaluation of Best Fit Hyper-Elastic Model for Sandwich Beam with SB Rubber Core. Int. J. Sci. Eng. Res..

[3] Yu F., Lu A., Lu J., Wang Z., Zhang Q., Geng C., Li Z. (2019). Effect of phenyl content, sample thickness and compression on damping performances of silicone rubber: A study by dynamic mechanical analysis and impact damping test. Polym. Test..

[4] Ge C., Rice B. (2018). Impact damping ratio of a nonlinear viscoelastic foam. Polym. Test..

[5] Chandran V., Nagarajan L., Thomas M.R. (2018). Evaluation of vibration damping behavior of different sizes of waste tyre rubber in natural rubber composites. J. Compos. Mater..

[6] Zorion K. (2016). Dynamic Stiffness and Damping Prediction on Rubber Material Parts, FEA and Experimental Correlation. Ph.D. Thesis.

[7] Sunali, Mago J., Negi A., Pant K., Fatima S. (2022). Development of natural rubber-bamboo biochar composites for vibration and noise control applications. J. Clean. Prod..

[8] Rahmani M., Adamian A., Hosseini-Sianaki A. (2021). Effect of Waste Ground Rubber Tire Powder on Vibrational Damping Behavior and Static Mechanical Properties of Polypropylene Composite Plates: An Experimental Investigation. J. Mater. Eng. Perform..

[9] Hassan M., Elsayed A., El-Souhily B., Elgamal H., Elshabasy M.M. (2023). Investigating the effect of augmenting the anti-roll-bar with a torsional-dynamic-absorber on the handling-stability and the ride-comfort of the off-road-vehicles. Alex. Eng. J..

[10] Dobrotă D. (2005). Some considerations regarding the constitutive equations used during the study of mincing rubber waste without insertion. Mater. Plast..

[11] Dobrotă D. (2015). Vulcanization of rubber conveyor belts with metallic insertion using ultrasounds. Procedia Eng..

[12] Xu L., Ni H., Tian Y., Sun D., Chen Z., Jin H. (2023). Multi-scale analysis of damping characteristics of dry mixed rubberized porous 2 asphalt mixtures for tire-pavement noise reduction. J. Clean. Prod..

[13] Li H., Long W.J., Khayat K.H. (2023). Efficient recycling of waste rubber in a sustainable fiber-reinforced mortar and its damping and energy dissipation capacity. Cem. Concr. Compos..

[14] Mei X., Sheng Q., Cui Z., Zhang M., Dias D. (2023). Experimental investigation on the mechanical and damping properties of rubber-sand-concrete prepared with recycled waste tires for aseismic isolation layer. Soil Dyn. Earthq. Eng..

[15] Zhu X., Miao C., Liu J., Hong J. (2012). Influence of crumb rubber on frost resistance of concrete and effect mechanism. Procedia Eng..

[16] Dharmaraj M.M., Chakraborty B.C., Begum S., Natarajan R., Chandramohan S. (2022). Effect of nanoclay reinforcing filler in nitrile rubber/polyvinyl chloride blend: Frequency response of dynamic viscoelasticity and vibration damping. Iran. Polym. J..

[17] Pistolas G.A., Anastasiadis A., Pitilakis K. (2018). Dynamic behaviour of granular soil materials mixed with granulated rubber: Influence of rubber content and mean grain size ratio on shear modulus and damping ratio for a wide strain range. Innov. Infrastruct. Solut..

[18] Suntako R. (2017). The rubber damper reinforced by modified silica fume (mSF) as an alternative reinforcing filler in rubber industry. J. Polym. Res..

[19] Doddamani M.R., Kulkarni S.M. (2012). Response of fly ash-reinforced functionally graded rubber composites subjected to mechanical loading. Mech. Compos. Mater..

[20] Dhamaraj M.M., Chakraborty B.C., Begum A. (2022). The effect of graphene and nanoclay on properties of nitrile rubber/polyvinyl chloride blend with a potential approach in shock and vibration damping applications. Iran. Polym. J..

[21] N A., Jakkamputi L.P., Gnanasekaran S., Thangamuthu M., Rakkiyannan J., Bhalerao Y.J. (2023). Dynamic Behavior Modeling of Natural-Rubber/Polybutadiene-Rubber-Based Hybrid Magnetorheological Elastomer Sandwich Composite Structures. Polymers.

[22] Song X., Wang W., Yang F., Wang G., Rui X. (2022). The study of natural rubber/polybutadiene rubber hybrid matrix-based magnetorheological elastomer. J. Thermoplast. Compos. Mater..

[23] Izquierdo M., Querol X. (2012). Leaching behaviour of elements from coal combustion fly ash: An overview. Int. J. Coal Geol..

[24] https://www.makeitfrom.com/material-properties/Plasticized-Flexible-Polyvinyl-Chloride-PVC-P.

[25] Dobrotă D., Petrescu V., Dimulescu C.S., Oleksik M. (2020). Preparation and Characterization of Composites Materials with Rubber Matrix and with Polyvinyl Chloride Addition (PVC). Polymers.

[26] Guignard M.I., Campagne C., Giraud S., Brebu M., Vrinceanu N., Cioca L.-I. (2015). Functionalization of a bamboo knitted fabric using air plasma treatment for the improvement of microcapsules embedding. J. Text. Inst..

[27] Dobrotă D. (2006). Experimental research regarding processing rubber waste with metallic insertions. Mater. Plast..

[28] Mirițoiu C.M., Stănescu M.M., Bolcu D. (2020). Researches Regarding the Mechanical Properties of a New Hybrid Vegetal Resin. Mater. Plast..

[29] Pashaeia S., Hosseinzadehb S., Siddaramaiahc B. (2019). Effect of carbon black and fly ash co-fillers content on mechanical and thermal behaviors of styrene butadiene rubber compounds. Eurasian Chem. Commun..

